# A Rare Continuum From Oral Submucous Fibrosis to Spindle Cell Squamous Carcinoma: A Case Report

**DOI:** 10.7759/cureus.100000

**Published:** 2025-12-24

**Authors:** Christeffi Mabel, Chamundeeswari P., Nivetha D., Ashwinthan M., Pooja P. A.

**Affiliations:** 1 Oral Medicine and Radiology, Chettinad Dental College and Research Institute, Chennai, IND

**Keywords:** aggressive form, lower gingivobuccal sulcus, oral sub-mucous fibrosis, rare case report, spindle cell squamous carcinoma

## Abstract

Spindle cell squamous carcinoma (SpCC) is a rare, aggressive sarcomatoid variant of squamous cell carcinoma (SCC) characterized by biphasic epithelial-mesenchymal morphology. Oral submucous fibrosis (OSMF), a potentially malignant disorder prevalent in South-Asia typically transforms into conventional SCC, while direct transformation into SpCC is exceedingly uncommon. We report the case of a 49-year-old male previously treated for OSMF who, four years later, developed a fissural defect in the right buccal vestibule without an exophytic mass. He returned one week later with numbness of the right lower lip and chin. An incisional biopsy demonstrated well-differentiated SCC, while definitive resection revealed predominant spindle-cell morphology consistent with SpCC (pT4aN2a). The histologic continuity between epithelial and spindle components suggests early epithelial dedifferentiation within the OSMF microenvironment, likely mediated by TGF-β-driven epithelial-mesenchymal transition. This case underscores the malignant potential of OSMF and highlights the importance of recognizing such atypical malignant transformations, which are vital for timely diagnosis and intervention.

## Introduction

Oral submucous fibrosis (OSMF) is a chronic, progressive, potentially malignant disorder (PMD) prevalent among South-Asian populations, associated primarily with areca nut [1]. Malignant transformation of OSMF into conventional oral squamous cell carcinoma (SCC) has been well documented, occurring in approximately 1.5-15% of cases [2]. In contrast, transformation into spindle cell squamous carcinoma (SpCC) is exceedingly rare, with only isolated reports available in the literature. SpCC is a distinct and aggressive variant of SCC characterized by a biphasic histological pattern comprising both epithelial and spindle components and is associated with a poorer prognosis due to its aggressive and high metastatic potential [3]. The exact mechanism of spindle transformation within OSMF remains unclear, but one proposed pathway is epithelial-mesenchymal transition (EMT), a biological process in which epithelial cells lose their characteristic polarity and cell-to-cell adhesion and acquire mesenchymal features such as increased motility and invasiveness [4]. EMT is a recognized mechanism in tumor progression and metastasis and has been implicated in the development of sarcomatoid carcinomas. In the context of OSMF, chronic inflammation, persistent mechanical trauma from fibrotic bands, and long-standing epithelial injury may act as initiating factors that promote EMT, leading to loss of epithelial polarity and acquisition of mesenchymal features [5]. Given OSMF’s unique fibrotic microenvironment characterized by sustained tumor growth factor-β (TGF-β) signaling, mechanical stiffness, areca-nut-induced genotoxicity, and altered mucosal stem cell markers, we hypothesize that OSMF may create permissive conditions for direct epithelial dedifferentiation into a spindle cell phenotype via EMT and cellular-plasticity pathways. Cellular plasticity pathways, which allow epithelial cells to reversibly shift between differentiated and dedifferentiated states, may further facilitate the emergence of spindle-shaped malignant cells. Such mechanisms provide a plausible explanation for direct epithelial dedifferentiation into a spindle cell phenotype without the requirement for a conventional squamous carcinoma precursor [6]. This case provides histologic evidence supporting such a transition and highlights the need for molecular profiling of PMD lesions to identify the risk of sarcomatoid transformation.

## Case presentation

**Figure 1:**
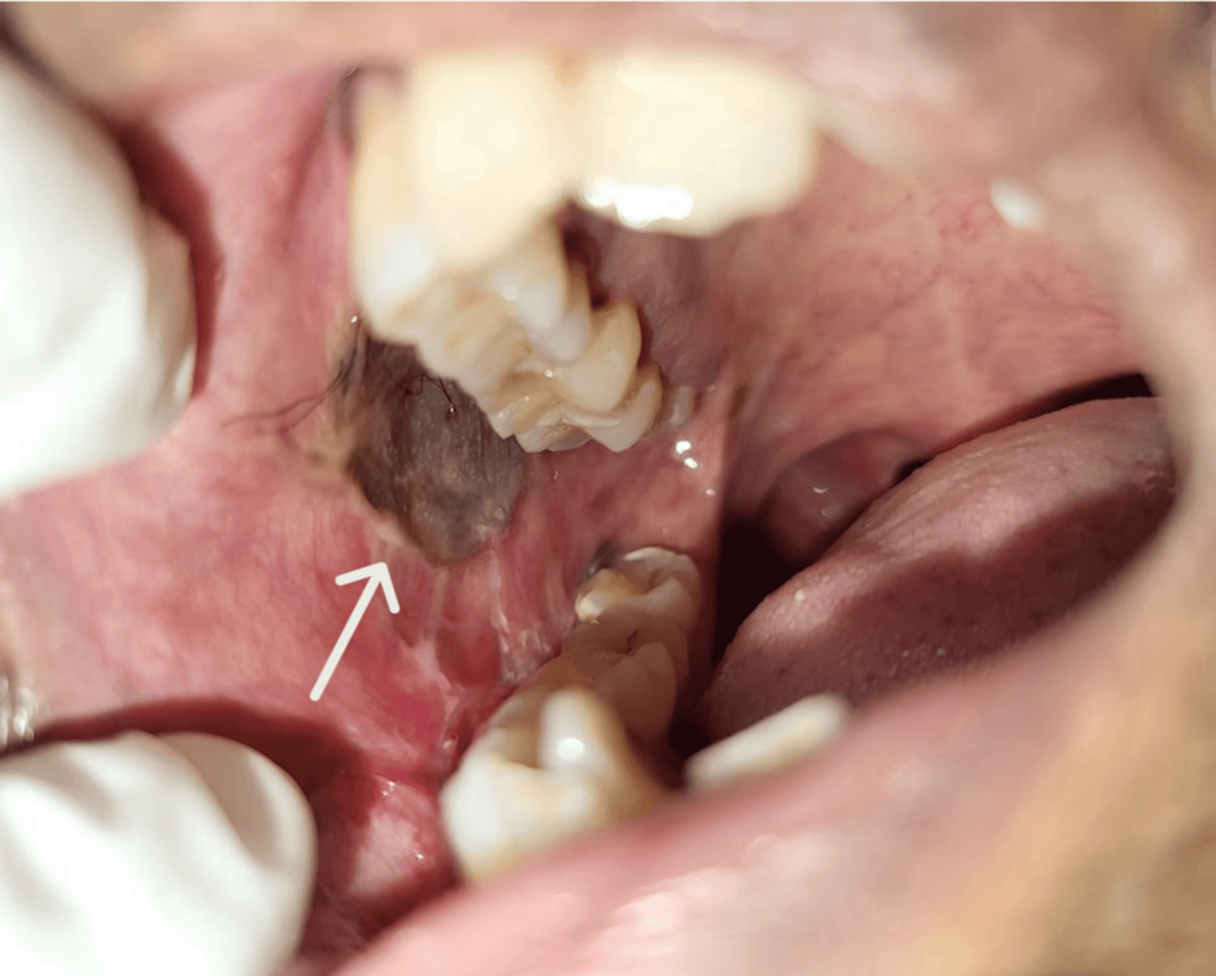
The site of the previously performed fibrotomy with myotomy and split-thickness skin grafting (SSG) (arrow).

A 49-year-old male presented to the Oral Medicine Outpatient Department with pain and swelling in the right lower jaw of two weeks’ duration. Four years earlier, he had experienced progressively restricted mouth opening and had undergone fibrotomy with myotomy and split-thickness skin grafting (SSG) (Figure 1).

Histopathological examination of the excised tissue at that time revealed stratified squamous epithelium lining with focal spongiosis. The underlying sub-epithelium showed fibrosis, chronic inflammatory infiltrate composed of lymphocytes, a few plasma cells, and a few congested blood vessels in the stroma. A few areas show hemorrhage. The following features were consistent with OSMF. No adjuvant or combination therapy was administered, and the patient reported no further treatment following the surgical procedure.

**Figure 2:**
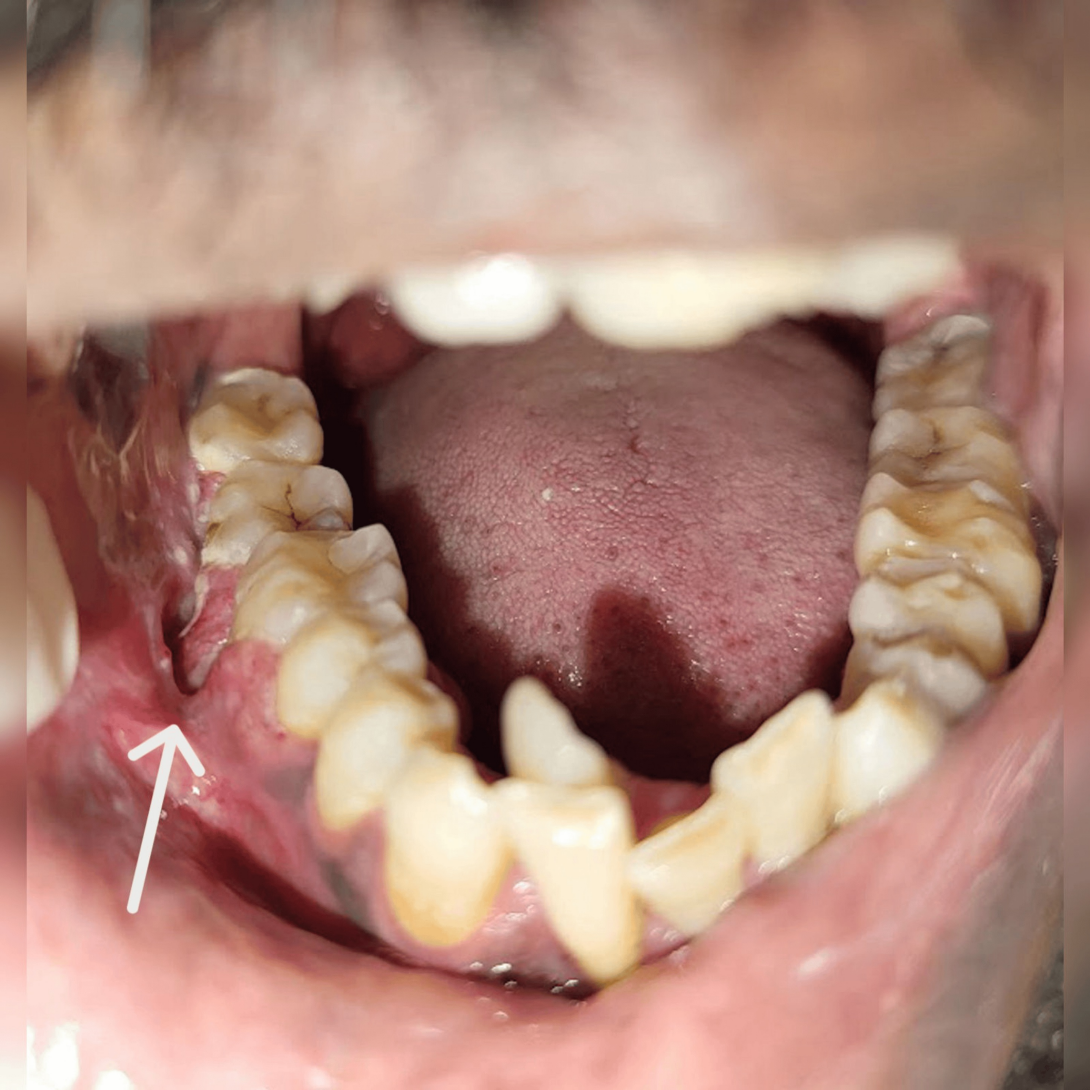
Fissural defect in the right gingiva-sulcus region (buccal vestibule) (arrow). A fissural defect measuring approximately 1 × 0.2 cm is noted in the right mandibular buccal vestibule in relation to tooth 46. It is oriented anteroposteriorly along 46 and extends mediolaterally within the vestibule. The attached gingiva, marginal gingiva, and interdental papilla show engorgement with loss of normal contour, appearing boggy and enlarged. A whitish, easily scrapable layer is observed adjacent to the fissured area, suggestive of surface sloughing or pseudo-membrane formation.

The patient had a history of betel-nut chewing for six years, along with cigarette smoking and alcohol consumption for over a decade, and has remained habit-free for the past five years following surgery. Extra-orally, a diffuse swelling was noted over the right mandibular body region. Intra-oral examination revealed a fissural defect in the buccal vestibule (Figure 2) in relation to 46 with grade II mobility of the tooth and adjacent mucosal induration. A provisional diagnosis of malignant soft-tissue neoplasm was suspected.

**Figure 3:**
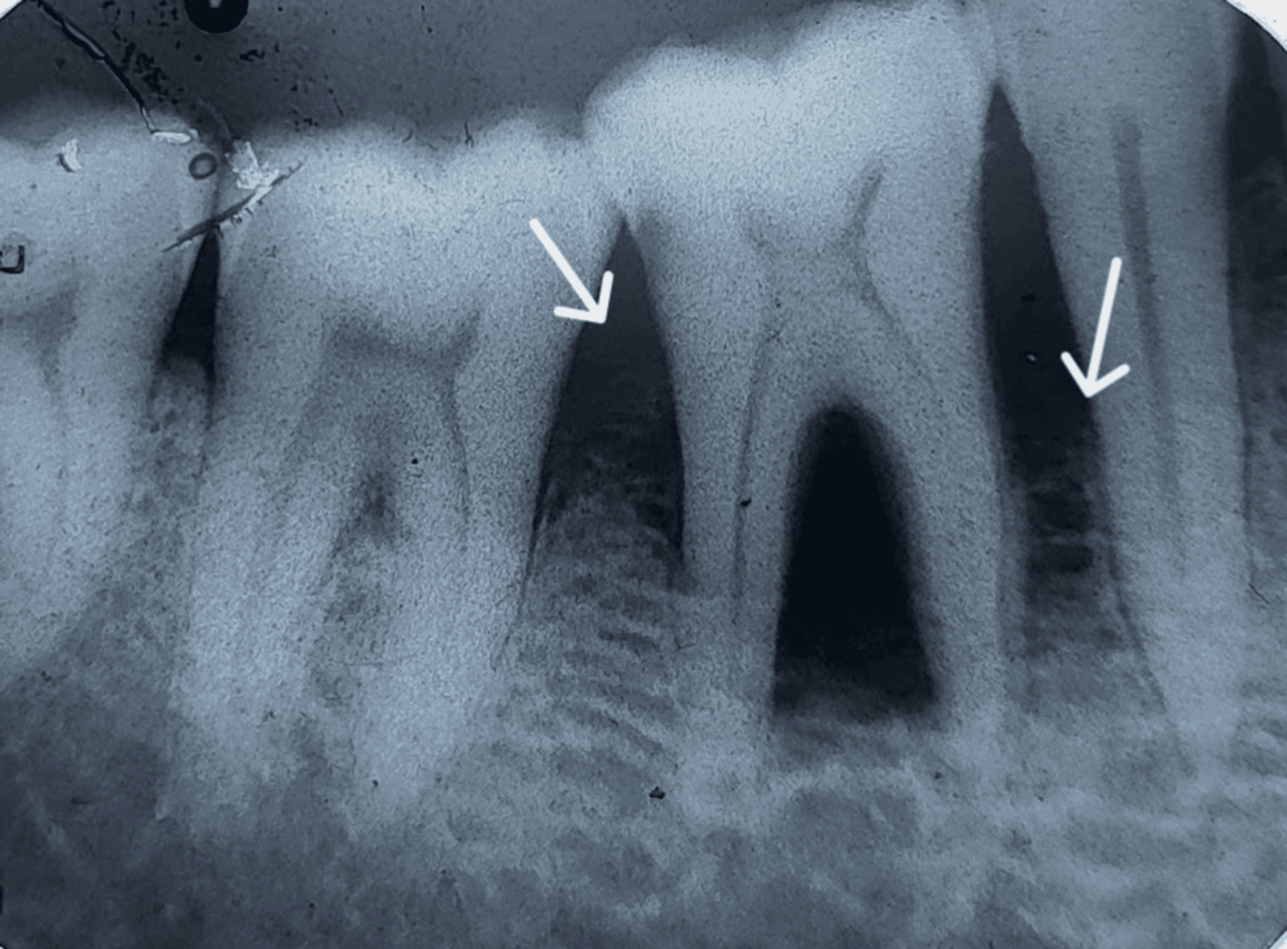
Intraoral periapical radiograph showing teeth 45, 46, and 47. There is horizontal alveolar bone loss in relation to teeth 45, 46, and 47, with interdental bone loss extending up to the cervical third between 46 and 47, and furcation involvement in 46 (arrows). Additionally, there is through-and-through bone loss in the furcation extending up to the apical third of the roots of 46.

An intraoral periapical radiograph (IOPA) revealed a scooped-out, through-and-through radiolucency in and around tooth 46 (Figure 3).

**Figure 4:**
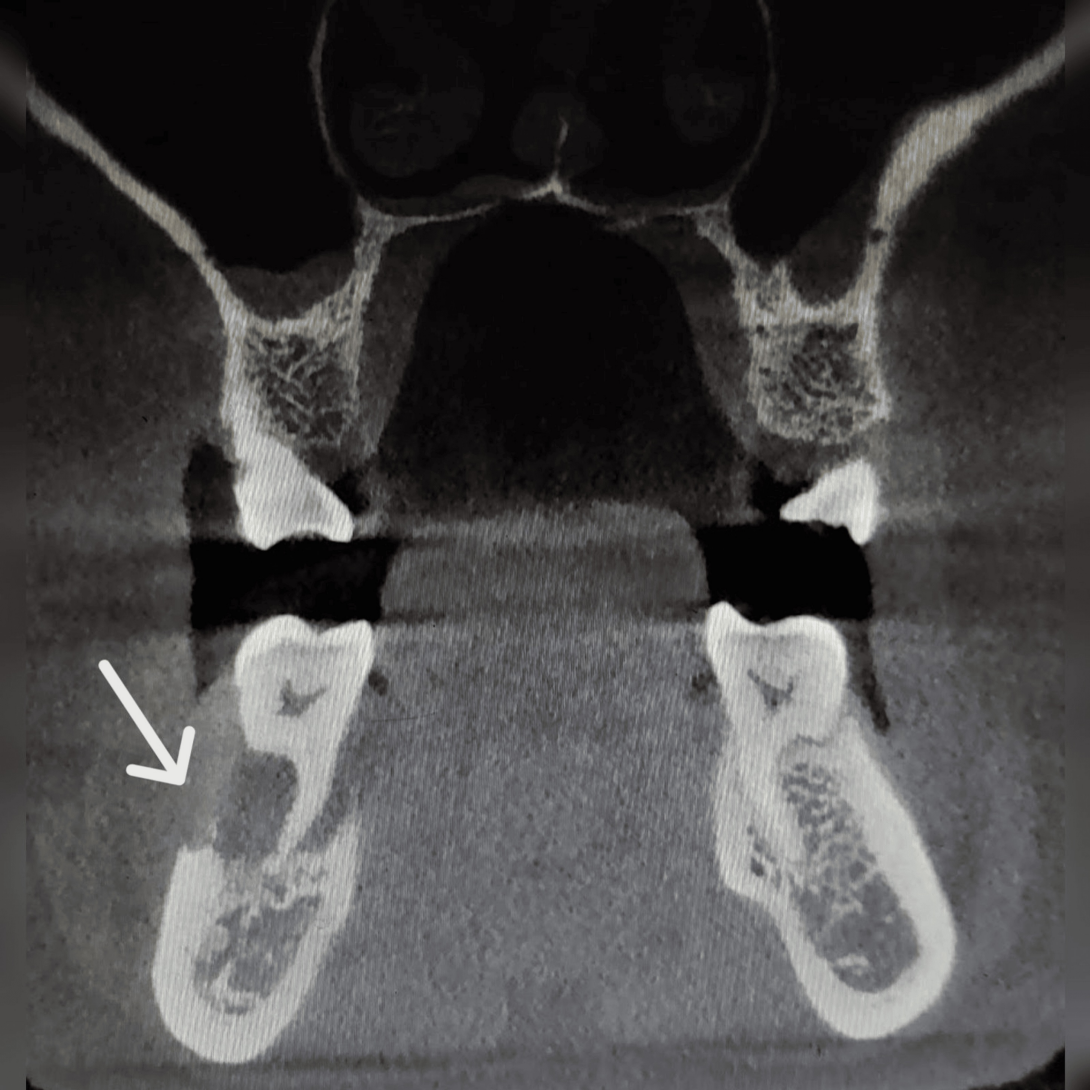
Coronal section at the level of tooth 46, showing destruction of the buccal and lingual cortical plates. The cone-beam CT (CBCT) coronal view of tooth 46 shows osseous destruction (arrow) involving both the buccal and lingual cortical plates, with significant loss of supporting alveolar bone. The borders of the affected cortical bone appear ragged and irregular, with no evidence of new bone formation.

**Figure 5:**
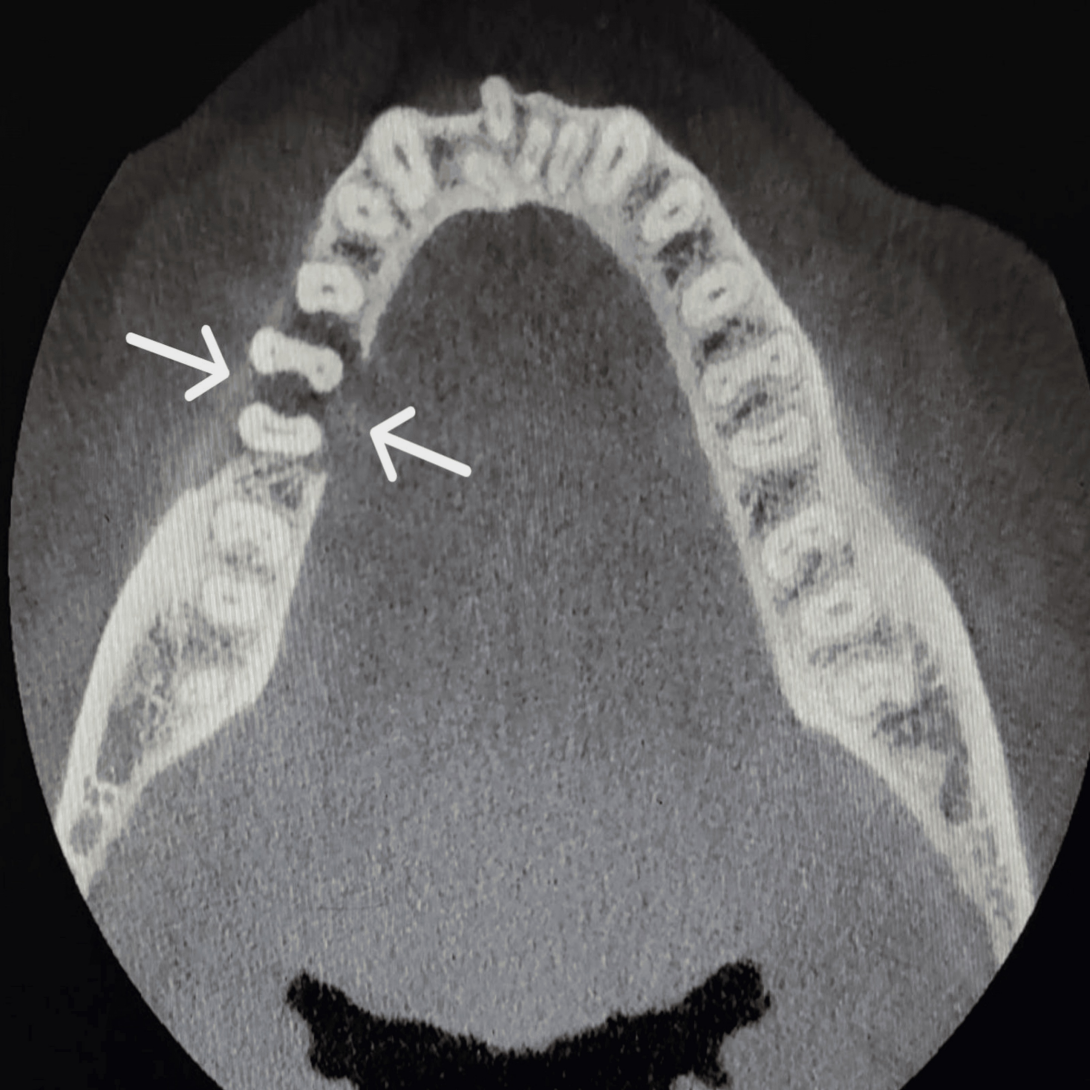
Axial section at the level of the cervical third of the root of tooth 46, showing erosion of the cortical plates (arrow). The image shows erosion of both the buccal and lingual cortical plates, with associated loss of supporting alveolar bone in relation to tooth 46.

Cone-beam CT (CBCT) demonstrated osseous destruction of both the buccal and lingual cortical plates, resulting in loss of supporting bone around tooth 46. The borders of the underlying cortical bone appeared ragged and showed no signs of bone formation, suggestive of a malignant process involving both soft and hard tissues. No evidence of root resorption was observed (Figures 4-5). 

**Figure 6:**
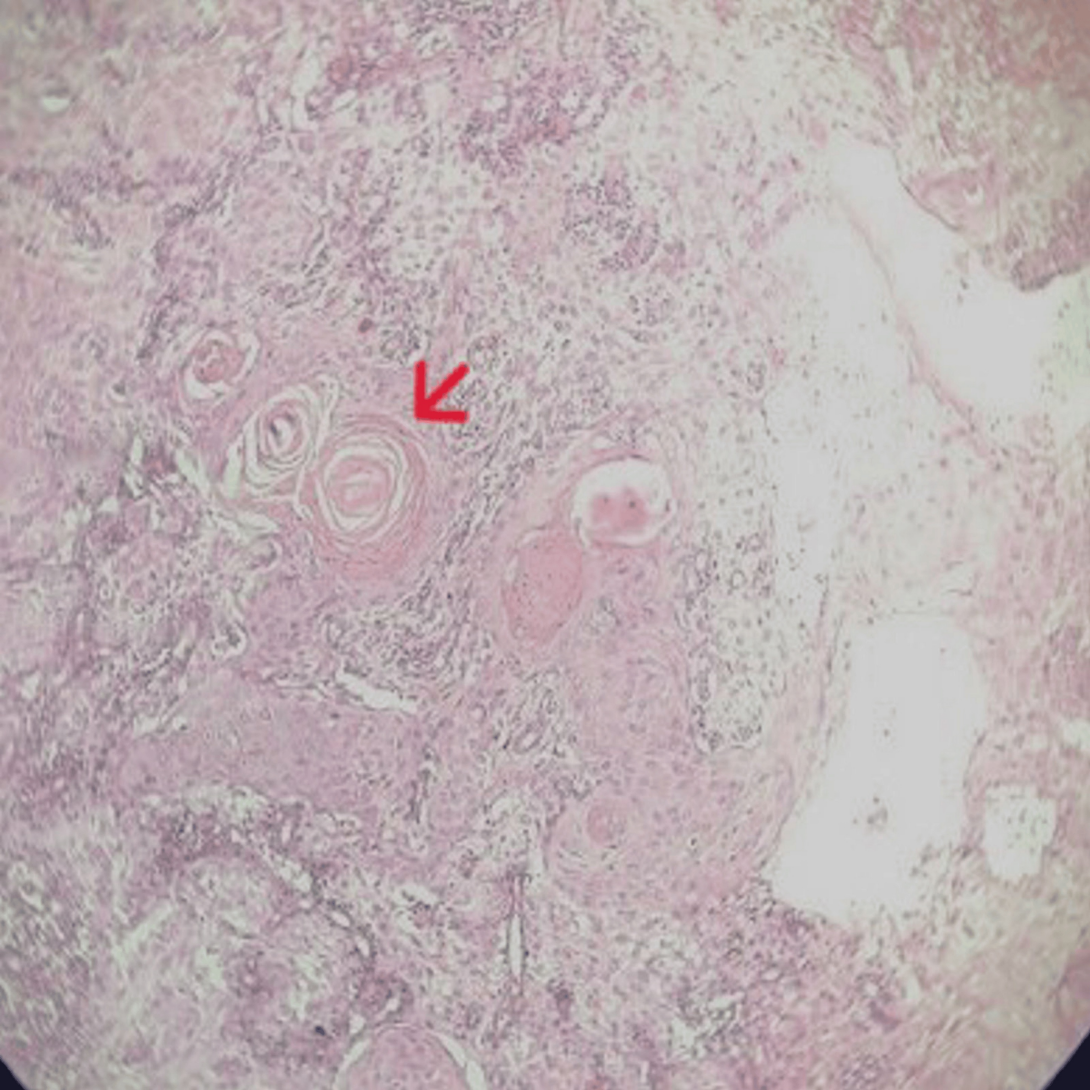
Histopathological image showing features of well-differentiated squamous cell carcinoma. Histopathological images show hyperplastic stratified squamous epithelium with basilar hyperplasia, acanthosis, cellular and nuclear pleomorphism, an increased nuclear-to-cytoplasmic ratio, and mitotic figures. Dysplastic squamous epithelial islands with prominent keratin pearl formation (indicated by a red arrow) are observed in a fibrocellular stroma, accompanied by dense inflammatory infiltrate and dilated capillaries.

An incisional biopsy was advised. The patient did not return for a week and subsequently presented with numbness of the right lower lip and chin, suggestive of Numb Chin Syndrome, a clinical indicator of mandibular nerve involvement. An incisional biopsy was performed, revealing hyperplastic stratified squamous epithelium with basilar hyperplasia, acanthosis, cellular and nuclear pleomorphism, an increased nuclear-to-cytoplasmic ratio, and mitotic figures. The presence of dysplastic squamous epithelium with pronounced keratin pearl formation is seen in the fibrocellular connective tissue stroma. Dense inflammatory cell infiltration and dilated capillaries are also observed. These histopathological features are suggestive of well-differentiated squamous cell carcinoma (Figure 6).

**Figure 7:**
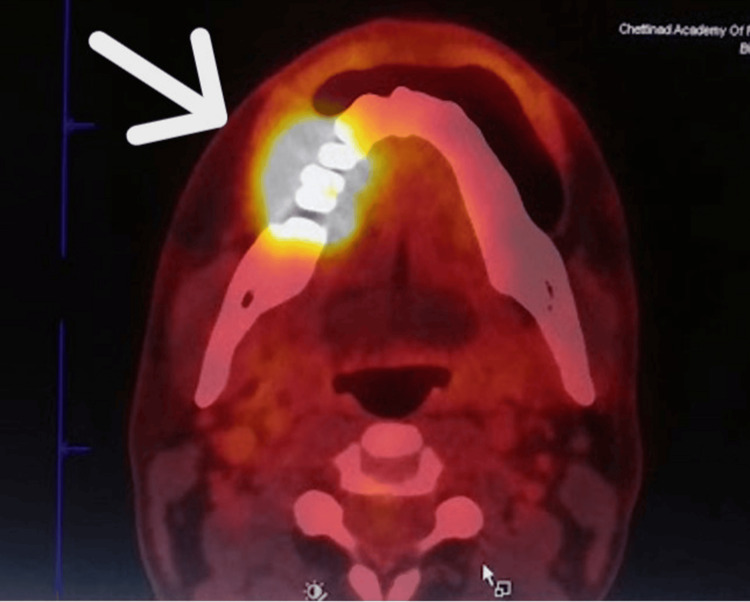
PET/CT showing increased metabolic activity in the region of tooth 46 (arrow), involving the gingivobuccal sulcus. A fluorodeoxyglucose (FDG)-avid hypodense lesion measuring approximately 3.2 × 2.0 cm (SUVmax 13.0) is noted in the right buccal mucosa and right gingivobuccal sulcus, with associated erosion of the mandibular cortices in the region of the right first molar.

**Figure 8:**
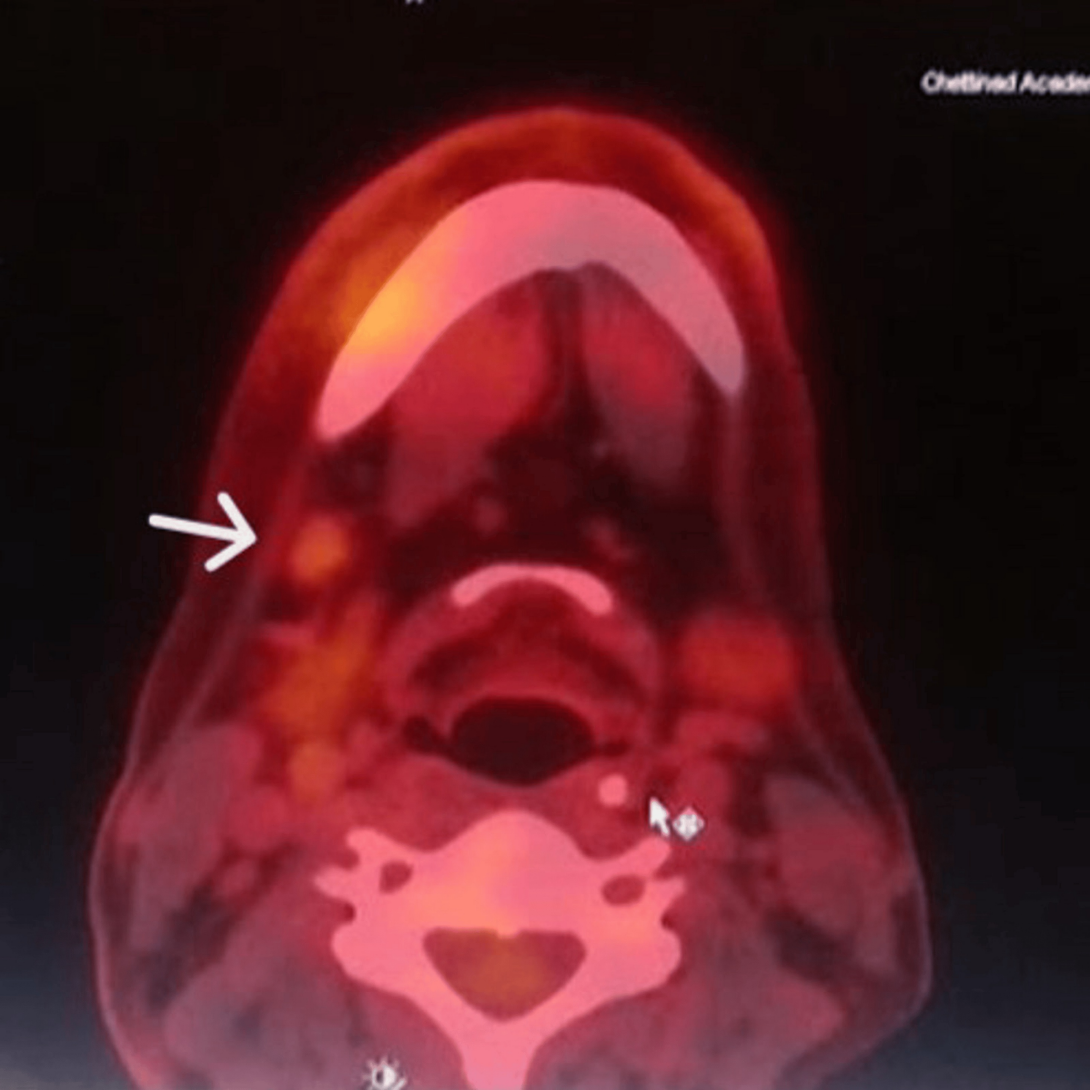
Mild metabolic activity is noted in a subcentimetric right level Ib cervical lymph node (arrow), suspicious for metastasis. A mildly fluorodeoxyglucose (FDG)-avid subcentimetric right level Ib cervical lymph node is observed, measuring approximately 0.7 × 0.7 cm, with an SUVmax of 2.0.

A positron emission tomography-computed tomography (PET-CT) scan was performed to rule out distant metastasis and revealed increased metabolic uptake confined to the right posterior mandibular region, consistent with a metabolically active primary malignancy. Mild uptake was also noted in the ipsilateral cervical lymph nodes, suggesting early nodal involvement (Figures 7-8).

Surgical management

A right composite resection with neck dissection was planned and executed.

Histopathology of the resected segment revealed a tumor size of 2.5 × 2.0 × 0.8 cm, diagnosed as a poorly differentiated SpCC with no lymphovascular invasion but with intra-tumoral perineural invasion. The tumor showed ulcerated stratified squamous epithelium with a neoplasm composed of sheets and nests of atypical squamous cells having moderate eosinophilic cytoplasm, enlarged vesicular nuclei with nucleoli, and keratin pearls, along with areas of spindle cell differentiation. Of the 10 lymph nodes examined, one node showed metastatic involvement measuring 0.8 cm, with the largest nodal metastatic deposit of 0.7 cm and extra-nodal extension (ENE) present. The tumor invaded the cortical bone of the mandible, and based on these features, the pathological classification was pT4aN2a, indicating a primary tumor invading bone with metastasis to a single ipsilateral lymph node showing ENE. Histopathological slide images were unavailable for review; therefore, only the findings described in the histopathology report were included, and representative photomicrographs could not be provided.

Intervention and follow-up

**Figure 9:**
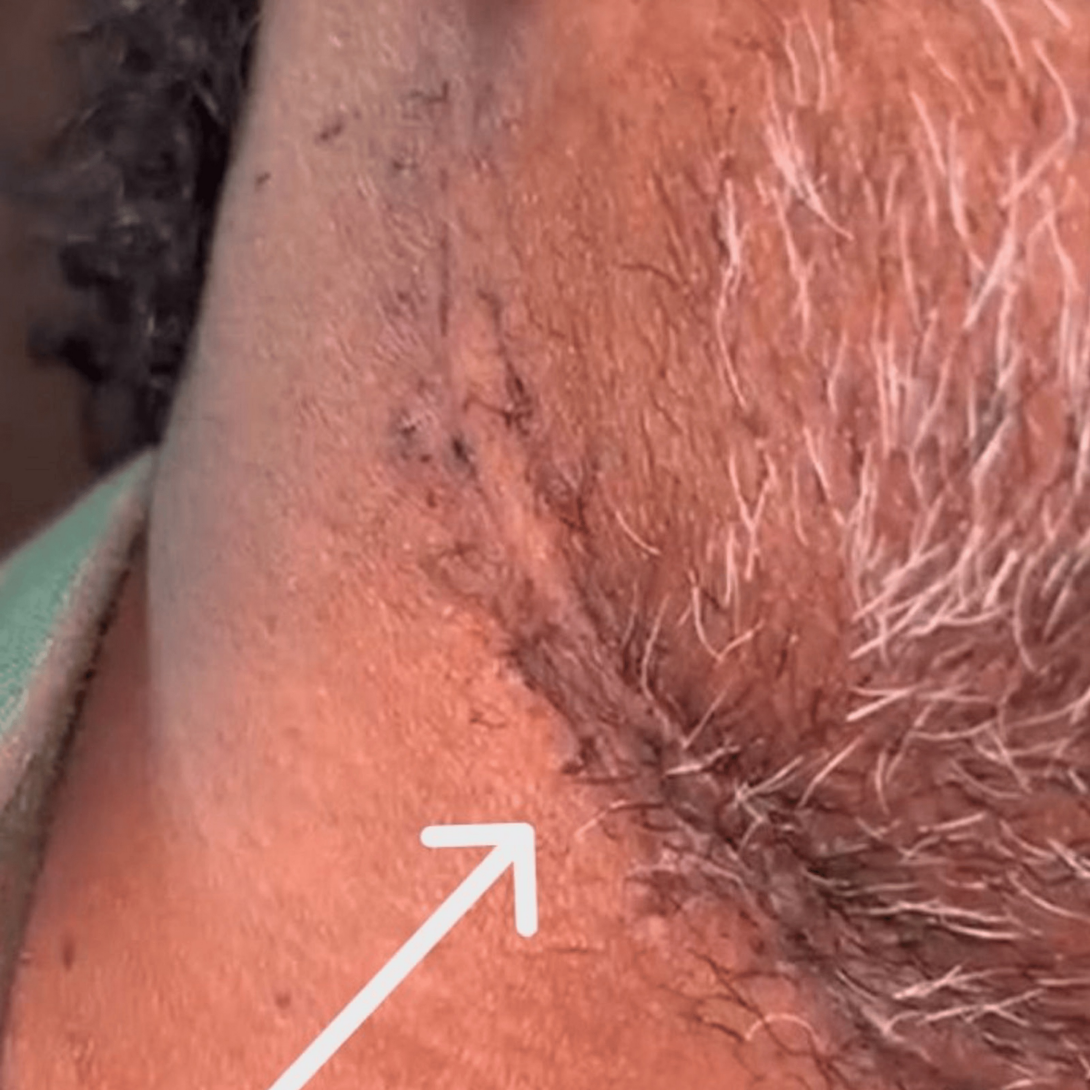
Postoperative extraoral image. Fourth-week follow-up showing extraoral healing (arrow) at the surgical site with satisfactory recovery.

The patient underwent surgical management at the end of June, followed by adjuvant concurrent chemoradiotherapy, which was initiated in early August. Radiotherapy was delivered over 33 fractions with concurrent cisplatin-based chemotherapy, administered with appropriate hydration and supportive medications. The postoperative recovery was uneventful (Figure 9). The patient has been on regular follow-up for the past two months at 15-day intervals. Currently, he reports restricted mouth opening, which is being managed with physiotherapy, along with oral burning sensation and difficulty in food intake, for which symptomatic management is provided.

## Discussion

SpCC represents a rare, aggressive variant of SCC characterized by a biphasic pattern of malignant epithelial and spindle cell components. These tumors are now recognized as monoclonal in origin, evolving from conventional SCC through dedifferentiation and EMT rather than arising de novo [1]. OSMF, a chronic, areca nut-associated fibrotic disorder prevalent in South Asia, is a well-established potentially malignant disorder. Telomerase reactivation in the chromosome plays a major role in the process of malignant transformation, most commonly into conventional SCC [7]. Direct transformation of OSMF into SpCC is exceedingly rare in the literature. To date, evidence is largely limited to isolated case reports and a few small head-and-neck series, with mandibular involvement representing only a negligible proportion. This underscores the marked paucity of data and highlights the need for further documentation.

In the present case, a 49-year-old male previously treated surgically for OSMF, after four years, developed a fissural defect without an exophytic mass or ulceration. The lesion was followed by Numb-Chin Syndrome, raising suspicion of perineural involvement. Incisional biopsy showed well-differentiated SCC, whereas the final resection specimen demonstrated predominant spindle-cell morphology (pT4aN2a). This histopathologic discordance aligns with the World Health Organization’s observation that small or superficial biopsies often sample only the epithelial component of SpCC, with the spindle component detected only in deeper or more extensive tissue [8].

The lesion’s spatial continuity with the previously fibrosed OSMF field, combined with chronic carcinogen exposure, supports field cancerization [9]. The histologic transition between epithelial and spindle areas further supports a dedifferentiation continuum rather than a secondary sarcomatoid transformation. Betel leaf-derived hydroxychavicol has been shown to induce COX-2 and prostaglandin E2 (PGE2) production in oral keratinocytes, potentially contributing to epithelial transformation [10]. Additionally, the OSMF microenvironment is rich in TGF-β activated by arecoline, a potent inducer of EMT, and is characterized by hypoxia, dense collagen deposition, and increased matrix stiffness [11]. These factors promote epithelial plasticity, cytoskeletal remodeling, and acquisition of spindle morphology. Formation of nitrosamines induces oxidative stress through interactions with DNA and proteins, contributing to carcinogenesis in both epithelial and submucosal tissues. Areca nut-derived alkaloids further generate reactive oxygen species and DNA adducts, accelerating genotoxic stress [10]. Together, these mechanisms create a biologically plausible pathway for direct epithelial dedifferentiation within OSMF, potentially bypassing the conventional exophytic SCC phase.

Although the monoclonal origin of SpCC from epithelial SCC has been confirmed through shared TP53 mutations and cytokeratin expression in both components, immunohistochemistry (IHC) and molecular studies were not performed in the present case, representing a limitation. Nonetheless, the morphologic continuity, clinicopathologic evolution, and early detection without distant metastasis reinforce the interpretation of early EMT-mediated dedifferentiation rather than secondary transformation. Future studies employing IHC (E-cadherin, vimentin, Snail/Twist, Ki-67, p53, β-catenin) or molecular clonality assays may further substantiate this pathway [12,13].

Unlike the cases reported by Rai et al. [3] and Rath et al. [14], where oral SpCC presented as rapidly enlarging exophytic masses, our patient exhibited only a fissural defect in the buccal vestibule without any proliferative growth. Occurring within a previously fibrosed OSMF field marked by rigidity and diminished vascularity, this unusual presentation suggests an infiltrative spread along fibrotic planes rather than outward tumor expansion.

Based on the present case, certain clinical features may serve as warning signs for atypical malignant transformation in patients with OSMF. These include the development of an unusual fissural defect with rapid progression in the absence of an exophytic mass, and early neurosensory symptoms such as numb-chin syndrome, which may suggest deep tissue or perineural involvement. However, these observations are derived from a single case and require validation through larger case series.

The combination of a subtle mucosal split and new-onset numb-chin syndrome may indicate early epithelial dedifferentiation with EMT-driven invasion, raising the possibility of atypical or direct sarcomatoid transformation within OSMF rather than progression from a conventional exophytic SCC.

## Conclusions

Clinically, this case highlights that malignant transformation in OSMF may not always manifest as an exophytic or ulceroproliferative lesion. The presence of fissural or submucosal defects within previously fibrosed areas should raise suspicion for malignancy, including atypical spindle cell differentiation. Timely biopsy and multidisciplinary evaluation are therefore essential. Recognition of such atypical presentations broadens the understanding of OSMF pathobiology and highlights the importance of long-term surveillance in patients treated for OSMF.
